# Cerebrospinal fluid efflux through dynamic paracellular pores on venules as a missing piece of the brain drainage system

**DOI:** 10.1002/EXP.20230029

**Published:** 2023-11-27

**Authors:** Yaqiong Dong, Ting Xu, Lan Yuan, Yahan Wang, Siwang Yu, Zhi Wang, Shizhu Chen, Chunhua Chen, Weijiang He, Tessandra Stewart, Weiguang Zhang, Xiaoda Yang

**Affiliations:** ^1^ Institute of Translational Medicine, The Affiliated Hospital of Qingdao University, College of Medicine Qingdao University Qingdao China; ^2^ The State Key Laboratories of Natural and Biomimetic Drugs and Department of Chemical Biology, School of Pharmaceutical Sciences Peking University Health Science Center Beijing China; ^3^ The National Institutes of Pharmaceutical R&D Co., Ltd. China Resources Pharmaceutical Group Limited Beijing China; ^4^ Department of Anatomy and Histology Peking University Health Science Center Beijing China; ^5^ State Key Laboratory of Coordination Chemistry, Coordination Chemistry Institute, School of Chemistry and Chemical Engineering Nanjing University Nanjing China; ^6^ Department of Pathology University of Washington School of Medicine Seattle Washington USA; ^7^ SATCM Key Laboratory of Compound Drug Detoxification Peking University Health Science Center Beijing China

**Keywords:** Aβ clearance, brain drainage pathway, dynamic asymmetric pores, perivascular space dilation, tight junctions

## Abstract

The glymphatic system plays a key role in the clearance of waste from the parenchyma, and its dysfunction has been associated with the pathogenesis of Alzheimer's disease (AD). However, questions remain regarding its complete mechanisms. Here, we report that efflux of cerebrospinal fluid (CSF)/interstitial fluid (ISF) solutes occurs through a triphasic process that cannot be explained by the current model, but rather hints at the possibility of other, previously undiscovered routes from paravenous spaces to the blood. Using real‐time, in vivo observation of efflux, a novel drainage pathway was discovered, in which CSF molecules enter the bloodstream directly through dynamically assembled, trumpet‐shaped pores (basolateral *ϕ*<8 μm; apical *ϕ* < 2 μm) on the walls of brain venules. As Zn^2+^ could facilitate the brain clearance of macromolecular ISF solutes, Zn^2+^‐induced reconstruction of the tight junctions (TJs) in vascular endothelial cells may participate in pore formation. Thus, an updated model for glymphatic clearance of brain metabolites and potential regulation is postulated. In addition, deficient clearance of Aβ through these asymmetric venule pores was observed in AD model mice, supporting the notion that impaired brain drainage function contributes to Aβ accumulation and pathogenic dilation of the perivascular space in AD.

## Graphical abstract

A novel drainage pathway through which cerebrospinal fluid (CSF)/interstitial fluid (ISF) molecules enter the bloodstream directly via dynamically assembled trumpet‐shaped pores on the walls of brain venules was discovered. An updated model for glymphatic clearance of brain metabolites and potential roles in the Alzheimer disease and some neurological disorders are discussed.

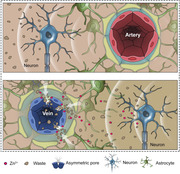

## 1 INTRODUCTION

Maintenance of brain homeostasis requires a delicate balance of the extracellular environment, necessitating fast clearance of interstitial fluid (ISF) solutes, especially metabolic wastes, by transfer to the blood through transport at the blood‐brain barrier (BBB), or through cerebrospinal fluid (CSF) drainage systems.^[^ ^1^ ^]^ Drainage via the glymphatic system, a recently‐recognized astrocyte‐driven process by which brain wastes including macromolecules and larger‐sized particles such as amyloid β (Aβ) and its soluble aggregates are eliminated,^[^ ^1^, ^2^, ^3^, ^4^, ^5^ ^]^ has been suggested to contribute to a large portion of clearance. The proposed^[^ ^4^ ^]^ pathway utilizes a loosening of the contacts between vascular endothelial and astroglial cells, primarily during sleep, to form a unique system of perivascular channels. CSF flows along the subarachnoid spaces into deep brain structures through the periarterial spaces and flushes through the cerebral parenchyma carrying the ISF solutes. Then the CSF/ISF collects in the paravenous spaces^[^ ^2^, ^3^, ^4^ ^]^ and is assumed to exit back into the subarachnoid spaces,^[^ ^6^ ^]^ where CSF is either absorbed into the dural venous sinuses via the arachnoid granulations/villi,^[^ ^6^, ^7^ ^]^ or drained through lymphatics associated with extracranial segments of the cranial nerves (such as meningeal lymphatic vessels) into cervical lymph nodes (CLNs).^[^ ^8^, ^9^ ^]^

Despite these advances in understanding of CSF/ISF clearance, several observations suggest that the above model is incomplete. The proposed process, for example, would allow the possible circulation of metabolic wastes inside the subarachnoid spaces. Additionally, questions remain regarding the anatomical structure of the perivascular spaces and the drainage pathways.^[^ ^10^ ^]^ For example, the conceptualization of paravenous spaces representing a temporal pool of ISF is not consistent with the observation that metabolic wastes like Aβ are deposited in pericapillary and periarteriolar membranes, but not in perivenular membranes.^[^ ^11^ ^]^ Similarly, abrogation of meningeal lymphatic vessels due to impaired VEGF‐C/D–VEGFR3 signalling reduced clearance of macromolecules, but not CSF water drainage, which should be coupled according to the current model.^[^ ^12^ ^]^ Moreover, when Aβ was injected into the parenchyma, its efflux in blood peaked quickly (half an hour after injection),^[^ ^13^ ^]^ sooner than the peak of CSF tracer in CLNs.^[^ ^2^, ^6^, ^10^, ^14^ ^]^ Together, these inconsistencies suggest the possibility that there may be additional, alternative routes for CSF/ISF solutes to exit the brain.

In considering new efflux routes between the perivascular spaces and the blood, we noted the following facts: (1) CSF flow is coupled to the brain's electrical activity,^[^ ^15^ ^]^ which may couple glymphatic pathway processes to sleep; (2) during neuronal activity, Zn^2+^ ions are released into the synaptic cleft, resulting in transient local Zn^2+^ concentrations of 100−300 μm, ^[^ ^16^ ^]^ and in some situations potentially reaching up to 600 μm;^[^ ^17^, ^18^, ^19^, ^20^, ^21^ ^]^ (3) Zn^2+^ may promote sleep efficiency and increase non‐rapid eye movement (NREM) sleep,^[^ ^22^, ^23^, ^24^ ^]^ a critical factor in activating the glymphatic system;^[^ ^4^, ^5^ ^]^ (4) Zn^2+^ at concentrations of >200 μm regulates the architecture of epithelial tight junctions (TJs), causing the formation of an asymmetric pore pathway favouring efflux, particularly for macromolecules.^[^ ^25^ ^]^ Consequently, we hypothesized that dynamic, asymmetric paracellular pores may exist on venules, undergoing regulation by brain electrical activity, and facilitating the flow of paravenous fluids directly into the venous blood flow, and sought to identify such pores using two‐photon laser scanning microscopy.

In the present work, we report the observation of dynamic formation of asymmetric trumpet‐like pores (basolateral *ϕ* <8 μm; apical *ϕ* <2 μm) on the walls of brain venules in mice. Through these asymmetric pores, CSF/ISF molecules were observed to directly converge into the bloodstream. As expected, addition of Zn^2+^ in perivenous flow could significantly promote formation of the outflow pores and facilitate brain clearance of the interstitial macromolecular solutes. Moreover, in Alzheimer disease (AD) model mice, reduced drainage of CSF/ISF containing elevated Aβ through the asymmetric pore path was observed, supporting the idea that deficient brain drainage contributes to the pathogenic dilation of perivascular space and Aβ accumulation in AD. Overall, our experimental evidence may provide one key piece for a more complete image of the brain glymphatic clearance system.

## 2 MATERIALS AND METHODS

### 2.1 Materials

Fluorescein isothiocyanate‐dextran 4 kDa (FD4) and Tetramethylrhodamine–dextran 70 KDa (TRITC70) were from Sigma Aldrich Tech Co. (USA). Eu_2_O_3_ (99.99%) and Diethylenetriaminepentaacetic acid (DTPA) were from Sinopharm Chemical Reagent Corp. (China). Bovine serum albumin (BSA) was from Amresco Inc. (USA). Artificial Cerebrospinal Fluid (ACSF) was from Leagene Corp. (China). Phosphate buffer saline (PBS) was from Hyclone (USA). Dimethylsulfoxide (DMSO) was from Sigma Aldrich Tech Co. (USA). Other reagents were of analytical grade.

### 2.2 Animals

C57BL/6 mice (male, 6–8 weeks old, SPF grade) were purchased from Peking University Health Science Center. APPswe/PS1dE9 (APP/PS1) transgenic mice and littermate negative C57BL/6 mice (male, 13–18 months old, SPF grade) were purchased from Model Animal Research Center of Nanjing University.

The mice were maintained and handled with the approval of Institutional Review Board for Laboratory Animal Care (Approval No. LA2017093) and fed in a barrier environment in Department of Laboratory Animal Science, Peking University Health Science Center. The mice were allocated randomly and group‐housed in a 12‐h light/12‐h dark cycle with ad libitum access to food and water. All experiments were performed in the light phase of the light/dark cycle. Pentobarbital sodium (1% in saline, 80 mg kg^−1^) was administered via intraperitoneal injection before experimental procedures. All efforts were made to keep animal usage to a minimum.

### 2.3 Preparation of Eu complexes and Zn^2+^ fluorescent sensor (NBD‐TPEA)

All Eu complexes were prepared according to the previously reported method.^[^ ^26^ ^]^ The stock solution of 0.01 m EuCl_3_ (pH 3.0) was prepared by dissolving 0.2760 g Eu_2_O_3_ in 5 mL of 3 m HCl and diluting to 100 mL with double distilled H_2_O.

#### 2.3.1 Eu‐DTPA

To a 0.01 m DTPA solution in Hank's balanced salt (HBSS; pH 7.0), 0.01 m EuCl_3_ was added drop‐wise until the appearance of a white sediment. The solution was kept at room temperature for 15 min, centrifuged (3 min, 10,000 × *g*), and the supernatant (Eu‐DTPA) was collected.

#### 2.3.2 Eu‐BSA

Briefly, the DTPA‐BSA conjugates were prepared by adding 18 mg DTPAA dissolved in DMSO to BSA (50 mg) solution in 5 mL of 0.1 m phosphate buffer with vigorous stirring. The coupling reaction proceeded 3−4 h at room temperature to allow the reaction to complete. Then, 0.01 m EuCl_3_ was added drop‐wise until appearance of a white sediment to form Eu‐DTPA‐BSA (Eu‐BSA). After centrifugation (3 min, 10,000 × *g*), the supernatant (Eu‐BSA) was collected and applied to a PD‐10 desalting column (GE Health Care, USA) pre‐balanced with HBSS (pH 7.0). The elute was concentrated by centrifugal ultrafiltration (Amicon Ultra‐4). The amount of BSA was measured with an enhanced BCA protein assay kit. The bound Eu was measured by time‐resolved fluorescence as described in the previous method (fluorescent parameter: *λ*_ex/em_ = 340/616 nm; measurement window, 600−1000 μs).^[^ ^26^ ^]^

#### 2.3.3 Zn^2+^ fluorescent sensor (NBD‐TPEA)

NBD‐TPEA was synthesized as previously described.^[^ ^27^ ^]^ The stock solutions of 5 mm NBD‐TPEA was prepared by dissolving 13 mg NBD‐TPEA in 524 μL of DMSO and diluting to 4.17 mL with PBS. NBD‐TPEA is a visible light excitable Zn^2+^ fluorescent probe based on the nitrobenzoxadiazole fluorophore. The probe has a good zinc ion selective enhancement effect, which can bind Zn^2+^ in a ratio of 1:1 and emit fluorescence at 534 nm with 488 nm excitation. With good Stokes’ displacement and biocompatibility, it is suitable for the quantitative measurements of zinc ion concentration in vivo or in vitro.^[^ ^27^ ^]^

### 2.4 Pharmacokinetics of intra‐striatal Eu‐DTPA injection in brain

To determine the kinetics of Eu‐DTPA probe in brain, Eu‐DTPA probe was injected into striatum of C57BL/6 mice (*n* = 5) . Specifically, anesthetized mice were fixed in a stereotaxic frame and body temperature was kept at 37°C with a temperature‐controlled warming pad. A 33 GA needle was inserted via a small burr hole into the brain at the following coordinates: 0.22 mm caudal, 2.5 mm lateral, 3.5 mm ventral to bregma.^[^ ^2^ ^]^ After needle insertion, 30 min elapsed to allow the needle track to swell closed, avoiding leakage of fluorescent agents from the needle insertion point. 1.0 μL of Eu‐DTPA probe (dissolved in ACSF) was injected at a rate of 0.1 μL min^−1^ with a syringe pump (Harvard Apparatus). Then, 15 min, 0.5 h, 45 min, 1 h, 3 h, 6 h, 12 h, and 24 h after the administration, animals were immediately decapitated, the skull opened, dura removed and the brain harvested. The brains of mice were combined with 5 times their own mass of pre‐chilled deionized water and homogenized with a bullet blender (Gene Company Limited, Hong Kong). The homogenates were centrifuged at 5000 × *g* for 15 min and the supernatant was collected. Control animals were injected with sterile 0.9% saline following the same procedure.

The Eu content of brain samples was measured by time‐resolved fluorescence as described in the previous method (fluorescent parameter: *λ*_ex/em_ = 340/616 nm; measurement window, 600−1000 μs)^[^ ^26^ ^]^ and normalized to percent of total injected amount of Eu‐DTPA probe. Eu‐DTPA clearance from the brain was compared between groups by two‐way ANOVA.

### 2.5 Intra‐striatal Eu‐BSA injection followed by Zn^2+^ treatment

C57BL/6 mice (male, SPF grade) were prepared and allocated randomly into four groups with six mice per group: (I) Control; (II) Zn^2+^ (0.25 mm) group; (III) Zn^2+^ (0.5 mm) group; (IV) Zn^2+^ (1 mm) group.

Anesthetized mice were fixed in a stereotaxic frame and body temperature was kept at 37°C with a temperature‐controlled warming pad. A 30 GA needle was inserted into the cisterna magna, and 2 μL of normal ACSF, 0.25, 0.5 or 1 mm Zn^2+^ (dissolved in ACSF) were injected at a rate of 0.2 μL min^−1^ over 10 min with a syringe pump (Harvard Apparatus) in groups I–IV, respectively. 30 min after Zn^2+^ injection, Eu‐BSA (constituted in ACSF) was injected into the striatum and allowed to circulate for 30 min. A 33 GA needle was inserted via a small burr hole into the brain at the following coordinates: 0.22 mm caudal, 2.5 mm lateral, 3.5 mm ventral to bregma. After needle insertion, 30 min elapsed to allow the needle track to swell closed. 1.0 μL of Eu‐BSA was injected at a rate of 0.1 μL min^−1^ with a syringe pump (Harvard Apparatus) in all four experimental groups.

After 30 min, mice were decapitated, urine and blood were collected and the brain harvested and homogenized. Eu content was also measured by time‐resolved fluorescence as described in previously and normalized to percent of total injected Eu‐BSA probe. Eu‐BSA clearance from the brain and accumulation in the urine were compared by two‐way ANOVA.

### 2.6 Intracisternal FD4 or Zn^2+^ (+FD4) or Zn^2+^ fluorescent sensor injection in healthy wild‐type mice and in vivo fluorescence imaging

C57BL/6 mice (male, 6−8 weeks old, SPF grade, 6 mice per experiment) were utilized as healthy wild‐type (WT) mice. A craniotomy (2 × 2 mm in diameter) was made over the cortex of anesthetized mice. The dura was left intact, and the craniotomy was covered with ACSF and sealed with a glass coverslip. After the recovery period from cranial window surgery, the discharge of various fluorescence tracers into the blood were observed. The anesthetized mice were fixed in a stereotaxic frame and a 30 GA needle was inserted into the cisterna magna. 2 μL of FD4 tracer or Zn^2+^ (together with FD4) or Zn^2+^ fluorescent sensor (NBD‐TPEA), respectively, was injected at a rate of 0.2 μL min^−1^ over 10 min with a syringe pump (Harvard Apparatus). To visualize the cerebral vasculature, 0.1 mL BBB impermeable Tetramethylrhodamine–dextran 70 KDa (TRITC70) (MW 70 kD, 1% in saline) was injected intravenously immediately before imaging.

After intracisternal injection of fluorescence tracers, tracer movement into the cortex was recorded with a confocal laser scanning microscope (Leica microsystems CMS GmbH D‐35578 Wetzlar (DFC360 FX), Germany) with FITC‐channel (FD4 tracer) or *λ*_ex/em_ = 488/534 nm (Zn^2+^ fluorescent sensor) 512 × 512 pixel image acquisition, as described below.

### 2.7 Intracisternal FD4 injection in AD mice model and in vivo fluorescence imaging

APP/PS1 transgenic mice (male, 13−18 months old, SPF grade, *n* = 4−6) and littermate negative C57BL/6 mice (male, 13–18 months old, SPF grade, *n* = 4−6) were used to examine CSF dynamics in a context relevant to AD. The APP/PS1 mice over‐express the delta exon 9 variant of presenilin 1 (PS1) in combination with the Swedish mutation of β‐amyloid precursor (APP). The FD4 tracer brain clearance experiments in the mice were conducted as in healthy WT mice.

### 2.8 In vivo two‐photon laser scanning microscopy

For in vivo imaging, a craniotomy (2×2 mm in diameter) was made over the cortex 1 mm lateral and 0.5 mm posterior to bregma.^[^ ^2^ ^]^ The dura was left intact and the craniotomy was covered with ACSF and sealed with a glass coverslip. To visualize the vasculature, 0.1 mL of BBB impermeable TRITC70 (MW 70 kD,1% in saline) was introduced by intravenous injection immediately before imaging. An HCX APO L 20×/1.00 water immersion lens was used to image the cortex, from the surface to a depth of ≈300 μm. Excitation wavelength was 920 nm for TRITC70 and FD4, and emission was collected at 500−550 nm for FD4 and 575−625 nm for TRITC70. The cerebral vasculature was imaged in 512 × 512‐pixel frames from the surface to a depth of 300 μm with 0.5 or 1 μm z‐steps by two‐photon laser scanning microscopy. After intracisternal injection of CSF tracer, tracer movement into the cortex was conducted with dual‐channel (FITC and TRITC) 512 × 512‐pixel image acquisition.

### 2.9 Blood vessel pore visualization

The cerebral vasculature was first observed using charge coupled device (CCD) dynamic imaging to set the target positions for the following two‐photon scanning. Venule pores were observed at the positions where CSF tracer directly entered the brain blood vessels along paravenous spaces. Images of the pore sections were collected at 0.2, 0.5, or 1 μm intervals, and the diameter along the depth from the basolateral to apical was measured.

Quantitative analysis of dynamic pores was measured in all fields of the craniotomy (2 × 2 mm in diameter). Mean values were calculated from 4−6 venules per animal in each region of different experimental groups. In order to observe the dynamic conditions of pores on the venule, the specific vessels would be tracked to be repeatedly scanned with 0.5 or 1 μm *z*‐steps at 1‐min intervals for the duration of the experiment.

### 2.10 Statistical analysis

Data were expressed as mean ± standard deviation (SD). For experiments with two groups, unpaired student's *t*‐tests were performed to test means for significance. For experiments with more than two groups, differences were analyzed by two‐way analysis of variance (ANOVA) and *p* < 0.05 was considered as statistically significant. Statistical analyses were carried out using Origin 8.0 or SPSS.

## 3 RESULTS

### 3.1 A novel pore path on venule walls accounts for the rapid drainage of CSF‐ISF solutes in brain

**FIGURE 1:**
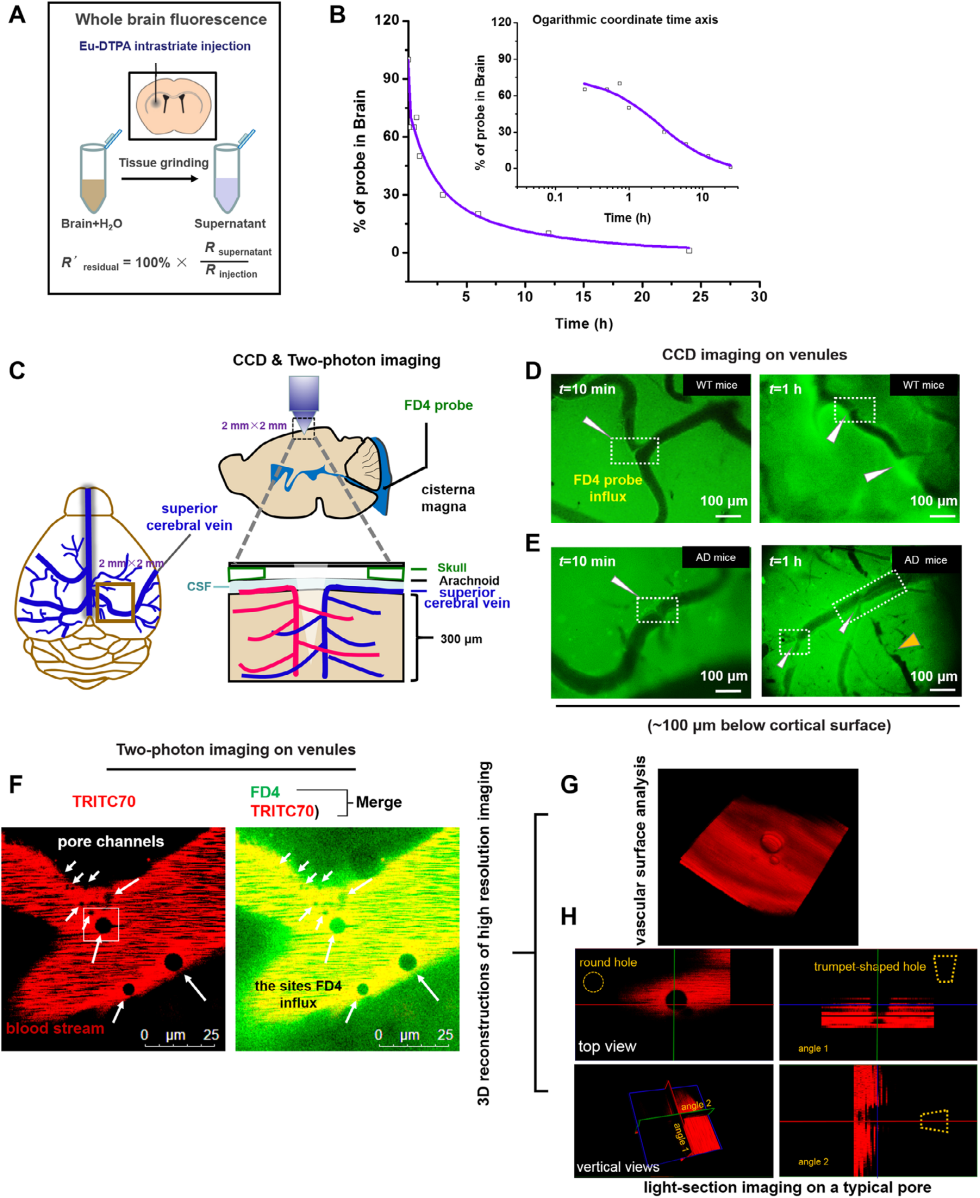
Rapid drainage of brain CSF‐ISF tracer through venule wall pores. (A) Small molecular weight BBB‐impermeable tracer, Eu‐DTPA complex, was injected into C57BL/6 mice striatum (*n* = 5) and the clearance from the brain parenchyma was evaluated. (B) The time course of drainage of intra‐striatal Eu‐DTPA probe. Data was fitted to a triphasic decay model using an Origin 8.0 program. (C) Illustration of experimental setup of in vivo observation of the dynamics of paravascular CSF tracers (FD4) flux into the mouse cortex and blood vessel. Imaging was conducted between 0 and 300 μm below the cortical surface. A superior cerebral vein branch vessel (in deep yellow rectangle) was used as the coordinate of depth to find cerebral venule vessels for observation. (D, E) Dynamic CSF fluorescence imaging (≈100 μm below the cortical surface) in healthy WT mice (D) and AD model mice (APPswe/PS1dE9 (APP/PS1) transgenic mice) with FD4 tracer injected into the mouse cisterna magna. White arrow indicates the spots for CSF entering the lumen of the blood vessel. Yellow arrow indicates the lumen of the blood vessel with stacking CSF fluorescent tracers. (F) Two‐channel zoom images on a typical site for CSF tracer entering the brain blood vessels from paravenous spaces as observed in (D and E). The pores were identified as black spots on the red channel (indicating blood flow and the surrounding vascular wall) overlapping with spots of the green (indicating CSF flow). The cerebral vasculature in healthy WT mice was visualized by intravenously injected TRITC70 and observed using two‐photon imaging confocal microscopy; (G) 3D reconstruction of high‐resolution light‐section images (red channel) for a typical pore; (H) the top and vertical views of 3‐dimension reconstructed pore in (G). Scale bars, 100 μm (D and E) and 25 μm (F).

The kinetic process for brain drainage was analyzed using europium‐DTPA (Eu^3+^‐DTPA), a BBB‐impermeable small molecular fluorescence tracer that is not a substrate of BBB efflux transporters, for quantitative measurement by taking the advantage of time‐resolved fluorescence to eliminate the background emission in biological samples.^[^ ^26^, ^28^ ^]^ After intra‐striatal injection of Eu^3+^‐DTPA (Figure 1A), the brain content of the fluorescent tracer was presented a rapid decline with time dependence, indicating a three phase process characterized by (Figure 1B): (1) very rapid clearance with a time constant (*τ*) of <5 min, contributing ≈25% of the tracer clearance under the test conditions; (2) fast drainage with a *τ* of 2.1 h, contributing ≈44% of tracer removal; and (3) slow drainage with a *τ* of 9.5 h for the remaining CSF‐ISF solute excretion (≈31%).

To our best knowledge, the fastest phase (*τ* < 5 min) does not match any of the currently known CSF drainage pathways. The existence of a more direct route for CSF‐ISF flux into the blood stream was investigated by fluorescence microscopy. The CSF‐ISF was visualized by infusion of fluorescein isothiocyanate–dextran‐4 (FD4, molecular size, 4 kD) into the cisterna magna, and the routes and time course of subarachnoid CSF flux into the brain parenchyma were recorded in real time through a closed cranial window in experimental mice conducted with anaesthesia (Figure 1C). The depth of our observation is 0–100 μm (the CCD imaging mode) and 0–300 μm (two‐photon scanning mode) beneath the pia mater. A superior cerebral vein branch vessel (Figure 1C) was used as the coordinate of depth to find a cerebral venule for observation. It was observed that CSF flux entered from the subarachnoid compartment into brain parenchyma resulting in a perivascular distribution around the venule, then flowed along the paravenous spaces, and drained directly into brain venule blood in both healthy (Figure 1D) and AD model (Figure 1E) mice. These movements are presented in Supplementary Videos 1–3. The large aggregate flow of FD4 tracers was easily seen around the walls of the blood vessels as in Supplementary Video 1. More careful observation revealed CSF tracers penetrating the vessel chamber through specific pore paths and moving along the inner side of vascular wall with blood flow as shown in Supplementary Videos 2 and 3. Compared with healthy WT mice, abundant tracer clogs were noted in the lumen of blood vessels near the pores in AD model mice (Figure 1E, indicated by yellow triangle).

To validate the exit of FD4 tracers into brain venule blood from the paravascular space, the pore paths in healthy WT mice were further observed using two‐photon imaging with additional visualization of the cerebral vasculature by intravenously injected tetramethyl‐rhodamine dextran 70 kDa (TRITC70). Considering the possibility that the observed results were the result of damage to the tissue induced by imaging, or were caused by the presence of rolling immune cells, the experiments were carefully conducted especially the two‐channel image dynamics (green for CSF flow and red for blood flow) were thoroughly compared. The results (Figure 1F–H) suggested existence of pores of differing sizes on the interface of CSF and blood flow (Figure 1F), which are characterized by spots of the green (indicating CSF flow) overlapping with black spots on the red (indicating blood flow and the surrounding vascular wall). To exclude the possibility of mistaking rolling cells or adhesion molecules as pores on the venule wall, a 3D image reconstruction was generated on a typical pore (Figure 1G). The different views of such 3D images (Figure 1H) clearly indicate an asymmetric trumpet‐like pore structure as the novel efflux pathway by which CSF‐ISF solutes are excreted into blood.

### 3.2 In vivo imaging reveals dynamic formation of asymmetric trumpet‐like pores on venule walls

**FIGURE 2:**
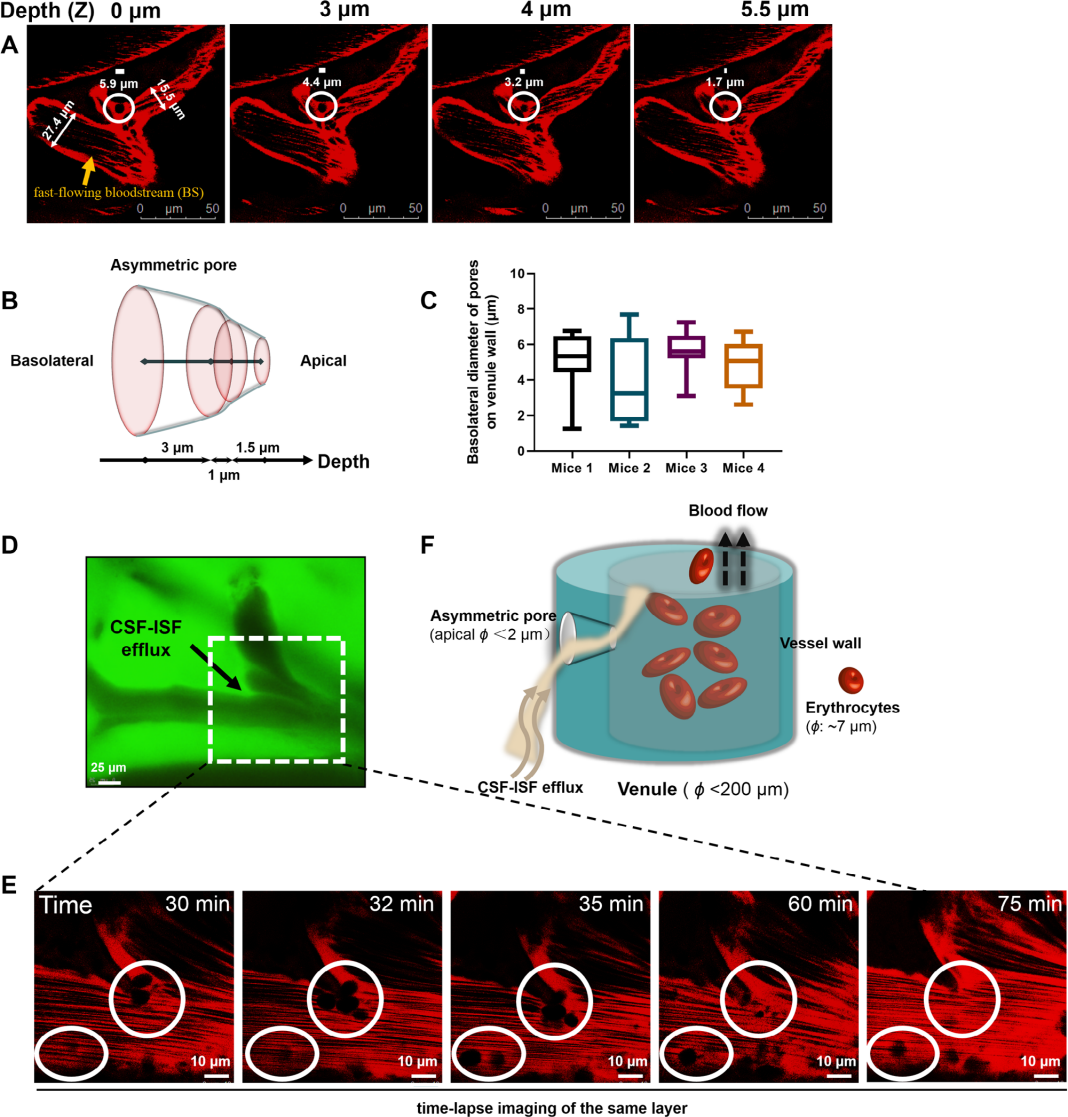
Dynamic asymmetric trumpet‐like pores on venule mediate direct entry of subarachnoid CSF‐ISF into blood vessels. The CSF/ISF and cerebral vasculature were visualized with FD4 and TRITC70, respectively. The morphological changes were observed with two‐photon and/or CCD dynamic imaging. (A) Light section images on a typical pore at different depths from the basolateral to apical side of vascular wall. The depth for first appearance of black “hole” at the basolateral side of venule is defined as zero. The pore diameters were 5.9, 4.4, 3.2 and 1.7 μm for the depth of 0, 3, 4, 5.5 μm, respectively. The yellow arrow indicates background of the fast‐flowing bloodstream (BS) in the veins; (B) reconstruction of the shape of the asymmetric trumpet‐like pore on venule observed in (A); (C) statistics of the basolateral diameter of pores on venule wall in the 2 mm×2 mm cranial window in four wild type (WT) mice; (D and E) temporal changes of pores on venule wall. A major site for CSF/ISF efflux was chosen from fluorescent imaging of CSF/ISF flow upon injection of FD4 tracer into cisterna magna of WT mice (white rectangle in (D)). The vessel images were taken at 30, 32, 35, 60, 75 min after injection; (F) schematic of CSF‐ISF flux through the asymmetric pores when moving along the paravenous space. Scale bars, 50 μm (A), 25 μm (D), and 10 μm (E).

Venule wall pores were further characterized using double labelling (TRITC70 and FD4) and two‐photon confocal microscopy. The region of CSF‐ISF flux was imaged at four depths, starting from the first appearance of a black “hole” at the basolateral side of venule (defined as depth = 0) to its disappearance in the background of flowing bloodstream (Figure 2A). 3D reconstruction of the pore shown in Figure 2B revealed an asymmetric structure 5.5 μm in depth, 5.9 μm in diameter on the basolateral side, and 1.7 μm in diameter on the apical side. The average basolateral diameter of imaged pores was 4.9 ± 1.8 μm (Figure 2C); accordingly, the apical diameter of the pores is expected to be <2 μm in average. Further statistical investigation revealed that the asymmetric pores could be observed in venules with vascular diameter of 15−200 μm.

Longitudinal observation over a 2 h period revealed dynamic formation of these asymmetric pores. As shown in Figure 2D,E, a cluster of pores (upper white circle) at a major site for CSF/ISF efflux site gradually shrank and disappeared over ≈1 h. Meanwhile, another pore emerged (lower white circle) at ≈30 min mark and remained stable for at least 30 min. This result suggested that these venule wall pore structures continuously assemble and disassemble, possibly under regulation of established inducers of vascular endothelial cell permeability. Together, these results suggest the dynamic process summarized in Figure 2F.

### 3.3 Zinc ions promote the formation of asymmetric venule pores and rapid discharge of macromolecules

**FIGURE 3:**
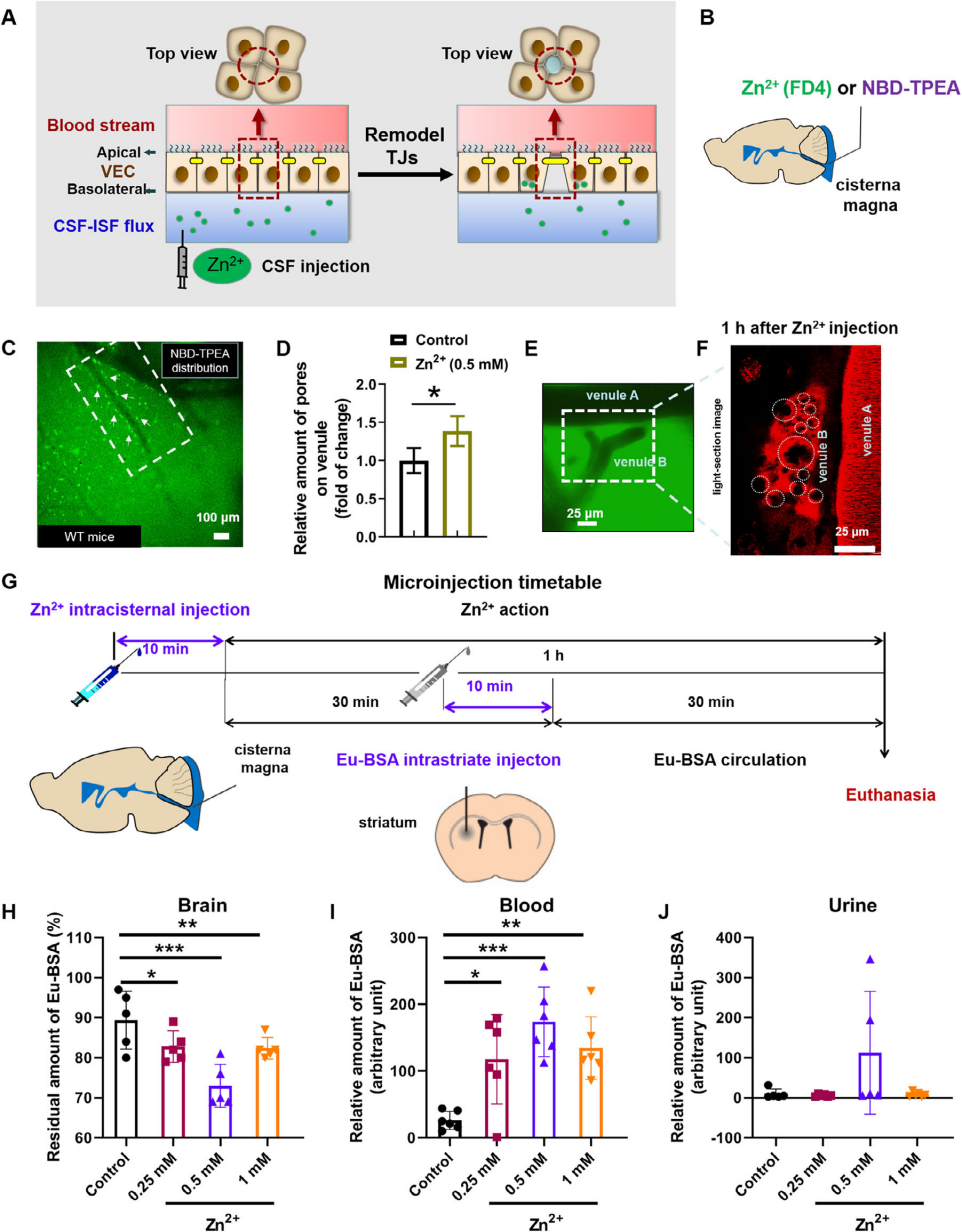
Zn^2+^ promotes pore formation and ISF and solute clearance from the brain. (A) Schematic of potential mechanism for formation of the asymmetric trumpet‐like pores on venule upon Zn^2+^ stimulation. Zn^2+^ was injected into the subarachnoid CSF and assumed to collect in paravenous space to exert influence on vascular endothelial cells (VECs) from the basolateral side to enhance tight junction (TJs) remodelling; (B) illustration of the movement of ventricular and subarachnoid Zn^2+^ (together with FD4) or Zn^2+^ probe (NBD‐TPEA) into the brain parenchyma; (C) visualization of Zn^2+^ with NBD‐TPEA fluorescence probe after intraventricular infusion for 5 min; (D) increase of relative number of dynamic pores on venule upon intracisternal injection of Zn^2+^ (0.5 mm) for ≈1 h. Data represented as mean ± SD (*n* = 4); (E, F) typical view of pores on venule (F) on a major CSF exit site (white rectangle in (E)) using two‐photon imaging. (G) Illustration of injection design of Eu‐BSA (striatum injection) and Zn^2+^ (intracisternal injection). The volume of all injections was 2 μL; (H, I, J) The relative amounts of (H) Eu‐BSA in brain, (I) peripheral blood and (J) urine after half hour of clearance upon treatment with different concentrations of Zn^2+^ (0.25, 0.5, 1 mm). Data represented as mean ± SD (*n* = 5−6). ^***^ *p* < 0.001, ^**^ *p* < 0.01, ^*^ *p* < 0.05 versus untreated control mice. Scale bars, 100 μm (C), 25 μm (E), and 50 μm (F).

Previously, high concentrations (>200 mm) of Zn^2+^ at the basolateral side of the Madin–Darby canine kidney (MDCK) cell monolayer have been demonstrated to induce the reconstruction of endothelial tight junctions (TJs) to form asymmetric trumpet‐like pores.^[^ ^25^ ^]^ Considering the potential transient local high concentration of Zn^2+^ in brain, especially during neuronal activity, we speculated a role of Zn^2+^ in regulating the formation of the trumpet‐like pores (Figure 3A).

To test this hypothesis, the spatial distribution of Zn^2+^ during paravascular CSF tracer movement into the pores was first observed using a Zn^2+^ fluorescence sensor (NBD‐TPEA) (Figure 3B). The result (Figure 3C) showed that Zn^2+^ ions were present in localized puncta along the vascular walls (highlighted by in rectangle in 3C). Secondly, the effect of intracisternal injection of Zn^2+^ on the relative quantity of dynamic pores on venules was assessed. The results (Figure 3D–F) showed that addition of Zn^2+^ (0.5 mm) significantly increased the number of asymmetric pores compared with controls in which ACSF only was injected (Figure 1F). Although the asymmetric pores may appear at any place along the venule wall, they seems to gather more densely in the divergence points of the blood venules (as for venule B), which is, interestingly, not dissimilar to the previously observed flow of fluid from the lung into nearby arteries at the apex of a branched blood vessel.^[^ ^29^ ^]^ Thirdly, the rate of discharge of brain interstitial macromolecular wastes upon Zn^2+^ treatment (Figure 3G) was evaluated. As shown in Figure 3H–J, significantly decreased retention of Eu‐BSA in the brain was observed in Zn^2+^‐treated animals, particularly within the 0.5 mm Zn^2+^ group; meanwhile the levels of Eu‐BSA in peripheral blood (Figure 3I), and secretion in urine (Figure 3J) were significantly increased. To exclude any possible damage of BBB or/and vascular walls, the rate of leakage of Eu‐BSA from blood to brain were tested; only marginal amounts (<0.25%) of marker leakage were found for either control or the 0.5 mm Zn^2+^ groups, confirming the integrity of BBB and vascular walls. Overall, CSF Zn^2+^ in appropriate concentrations promoted the formation of the asymmetric pores as well as rapid discharge of macromolecular metabolic wastes in brain.

### 3.4 Aβ burden beyond pore path clearance capacity may contribute to perivenous space dilation in AD model mice

The formation of Aβ plaques is one hallmark of AD pathogenesis. Aβ accumulation arises from an imbalance between production and clearance, beginning in the early stages of AD.^[^ ^1^ ^]^ A variety of systems have been suggested to be involved in clearance of extracellular Aβ deposits in the brain, including transportation by the BBB, the glymphatic system and the meningeal lymphatic vessels.^[^ ^1^ ^]^ Given our findings of a novel efflux pathway, we investigated the clearance of Aβ through asymmetric venule pores in AD model mice.

**FIGURE 4:**
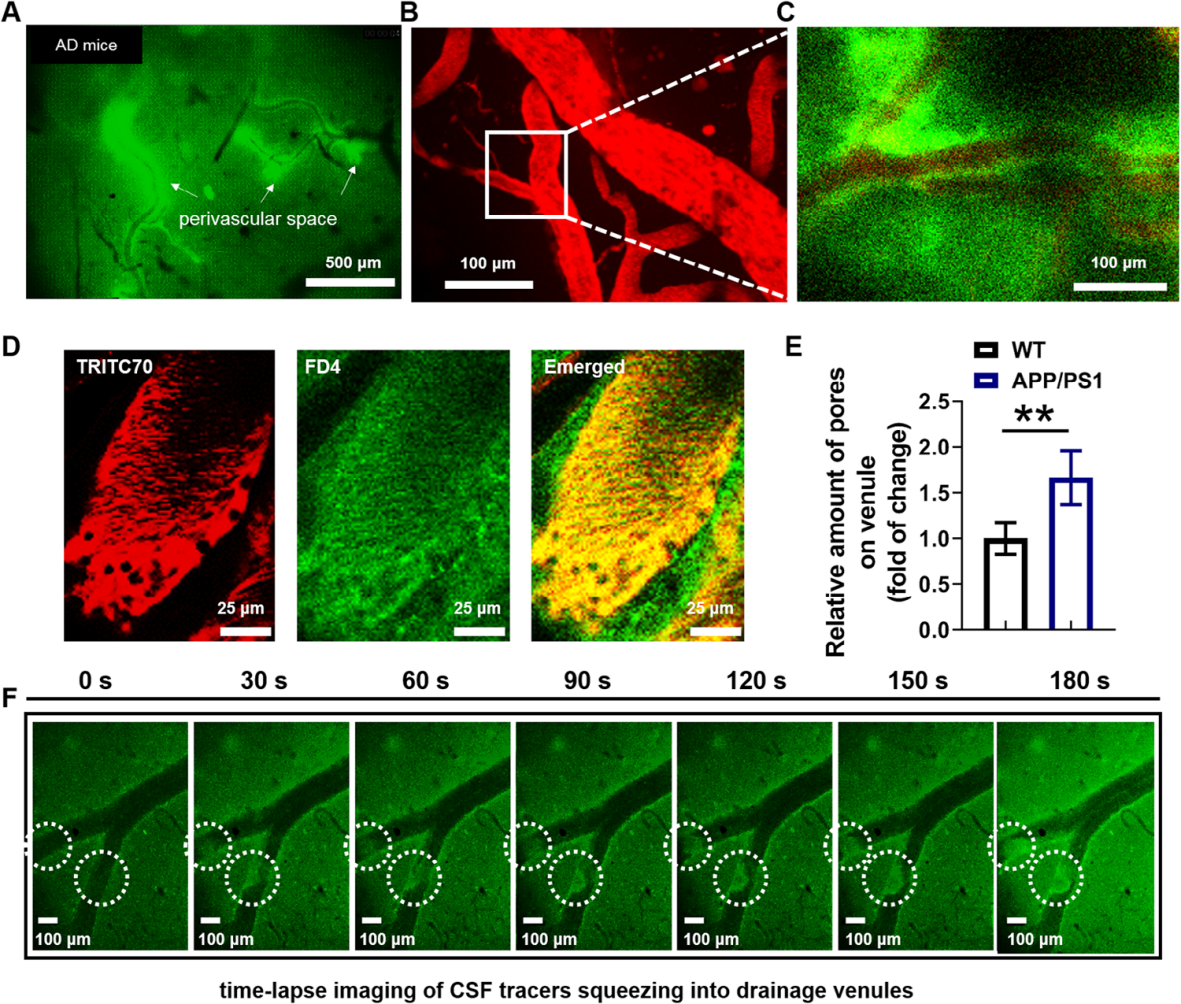
CSF/ISF solute clearance through venule pore paths in AD model mice. (A–C) Distribution of CSF fluorescence tracer (FD4) after intracisternal infusion for ≈1 h in AD model mice. (A) FD4 fluorescent tracers accumulated in the perivascular space of multiple blood vessels; (B) the cerebral vasculature visualized by intravenous TRITC70 (Red) and (C) in the selected area where observed CSF flux into vein blood (white rectangle in (B)), FD4 tracer (green) accumulated around the vasculature; (D) the two channel images by two‐photon confocal microscopy showed formation of the multiple pores through which CSF tracers flushed out; (E) the relative amount of dynamic pores on venule in AD mice increased significantly in comparison with the WT controls (^**^ *p* < 0.01). Data represented as mean ± SD (*n* = 4); (F) time course of CSF tracers squeezing into drainage venules. CSF tracers aggregate in chamber of vein around the site of entry (white circles) for more than 2 min. Scale bars, 500 μm (A), 100 μm ((B) and (C)), 25 μm (D), and 100 μm (H).

As shown in Figure 4A, after intracisternal injection in AD mice, the FD4 tracers were observed to accumulate in the perivenous areas alongside multiple blood vessels. It is noted that most of the FD4 tracer bands around brain venules in AD mice are noticeably wider than those in WT healthy mice. A closer look at the blood vessels and paravascular space (Figure 4B,C) revealed that the paravascular FD4 tracers seemed not to flow evenly around the vascular wall, but rather in a number of small, interconnected pools. Underneath the pools, multiple pores were observed on the venule walls, through which the CSF exited into venules (Figure 4D). The number of pores on venules of AD mice was around 60% greater than on venules of WT mice, consistent with our previous observation that AD mice showed a slightly increased rate of brain Aβ clearance.^[^ ^13^ ^]^ However, we also observed that the FD4 tracers accumulated near the vein, and remained in close proximity to the pore for some time (Figure 4F), suggesting that CSF in the paravenous pools may be highly viscous due to high content of Aβ.

The high viscosity of high Aβ‐content CSF is further demonstrated in Figure 4F and Supplementary Video 4. While in the healthy mice CSF flux carrying the FD4 tracers forms smooth streams into the blood through the asymmetric pores (Supplementary Videos 2,3), the CSF flux in AD model mice is slower, as if flow into the blood vessel chamber is prolonged, at times even remaining at the blood vessel entry point for tens of seconds (Supplementary Videos 5,6), possibly due to a temporary disruption of blood flow, before the tracer is finally flushed away (Supplementary Video 5). Remarkably, even once the blood flow was fully recovered (Supplementary Video 6), the tracer appeared to be partially retained, with an uneven, “gurgling” flow near the wall. Therefore, despite increased numbers of pores in AD model mice, the viscous, high Aβ‐content CSF could exceed the drainage capacity of even pore‐enriched vessels, which may lead to CSF retention that could be associated with the perivascular space dilation, a known indicator of AD progression^[^ ^30^ ^]^ due to declined glymphatic clearance prior to the formation of amyloid plaques.^[^ ^31^ ^]^

## 4 DISCUSSION AND CONCLUSION

It is likely that CSF/ISF drainage occurs through multiple pathways. To estimate the contribution of each of the possible pathways, we analyzed the kinetic drainage process of a small molecular weight, BBB impermeable fluorescent lanthanide probe (Eu^3+^‐DTPA), which is rather stable to metabolic degradation in CSF and is one of the most sensitive indicators in time‐resolved fluorescence spectroscopic measurements.^[^ ^26^, ^28^ ^]^ The results (Figure 1A,B) showed a three‐phase process, indicating a similar number of likely mechanisms. The medium rate (*τ* ≈ 2.0 h) matches well with that of drainage through the dural lymphatics in most reports;^[^ ^12^ ^]^ The slowest (*τ* ≈ 10 h) may suggest possible existence of reservoirs or pools in the brain, in which waste products are temporarily stored, and which require further investigation. However, the rapid phase (*τ* < 5 min) requires explanation including a new mechanism with a shorter distance or/and more direct discharge to the blood.

In observing the dynamic process of subarachnoid CSF influx into brain parenchyma, we identified a new pore‐based pathway for fluorescence CSF/ISF tracers to directly enter the brain blood vessels (Figure 1C–E; Supplementary Videos 1–3). The direct route of CSF entry is supported by the observation that the tracers were seen to inject from the paravascular spaces into the venous blood through a point on venule wall (Figures 1E and 4). Further careful observations and 3D reconstruction (Figures 1F–H and 2) revealed that the pores show an asymmetric trumpet‐like shape and dynamically form/vanish on venule walls. As the pore formation is a function of living vascular endothelial cells, this structure would be challenging or impossible to observe by delayed and in vitro analysis such as traditional electron microscopy. Another limitation of the present study was that the dynamic formation of the pore structure might be influenced by the associated brain surgery, although BBB was shown to be intact and the injection was confirmed to not significantly affect the periarterial CSF flow of cerebrospinal fluid.^[^ ^32^ ^]^ Therefore, full characterization of the asymmetric pore paths would be appropriate in the future using new real‐time and non‐invasive in vivo imaging methods in combination with fluorescence protein transgenic animal models.

The mechanism by which such formation of venule pores occurs, while avoiding induction of cerebral haemorrhage is a major question. Theoretically, the CSF‐ISF flow through the pore paths is a one‐way process (from paravenous space to blood), maintained by the following factors: (1) the trumpet‐like shape of the pore favours solute movement from the larger diameter basal side to the smaller apical side, which is similar to asymmetric ion transport by tapered nanopores;^[^ ^33^ ^]^ (2) according to the Bernoulli principle,^[^ ^34^, ^35^ ^]^ there exists a hydrodynamic pressure difference from the static ISF to the flowing blood; (3) CSF oscillation dynamics^[^ ^15^ ^]^ may generate additional hydrostatic pressure from brain parenchyma to blood; (4) the apical size (<2 μm) of the pores is small, restricting the bilateral passage of blood cells. In fact, only marginal leakage of Eu‐BSA markers was observed from the blood side. Therefore, the unidirectional CSF‐ISF flow would preclude reverse entry of any blood molecules. Identification of further precise details on how the CSF‐ISF flow changes through the pore channel in pathological conditions presents an intriguing topic of future research.

Although additional details regarding the regulation of dynamic pore formation remain to be discovered, we speculate that this process would be modulated by a variety of factors in the neuronal electric activities coupled with the hemodynamics and CSF oscillations in sleep^[^ ^15^ ^]^, amongst which Zn^2+^ may play an important role, as suggested by the current results (Figure 3). Neuronal activity during CSF oscillations may involve the release of pulsatile transient local high concentrations of Zn^2+^ (observed herein as fluorescent spots in Figure 3C) that participate in inducing remodelling of TJs of vascular endothelial cells to form dynamic pore paths.^[^ ^16^, ^25^ ^]^ In certain cases, it is interesting to observe that the pulse of ISF injection into venous blood seemed to couple with the vascular pulsation (Supplementary Video 7), supporting that the pulsatile inflow of CSF coupled to hemodynamic processes^[^ ^15^ ^]^ may also provide an additional driving force for CSF movement.

**FIGURE 5:**
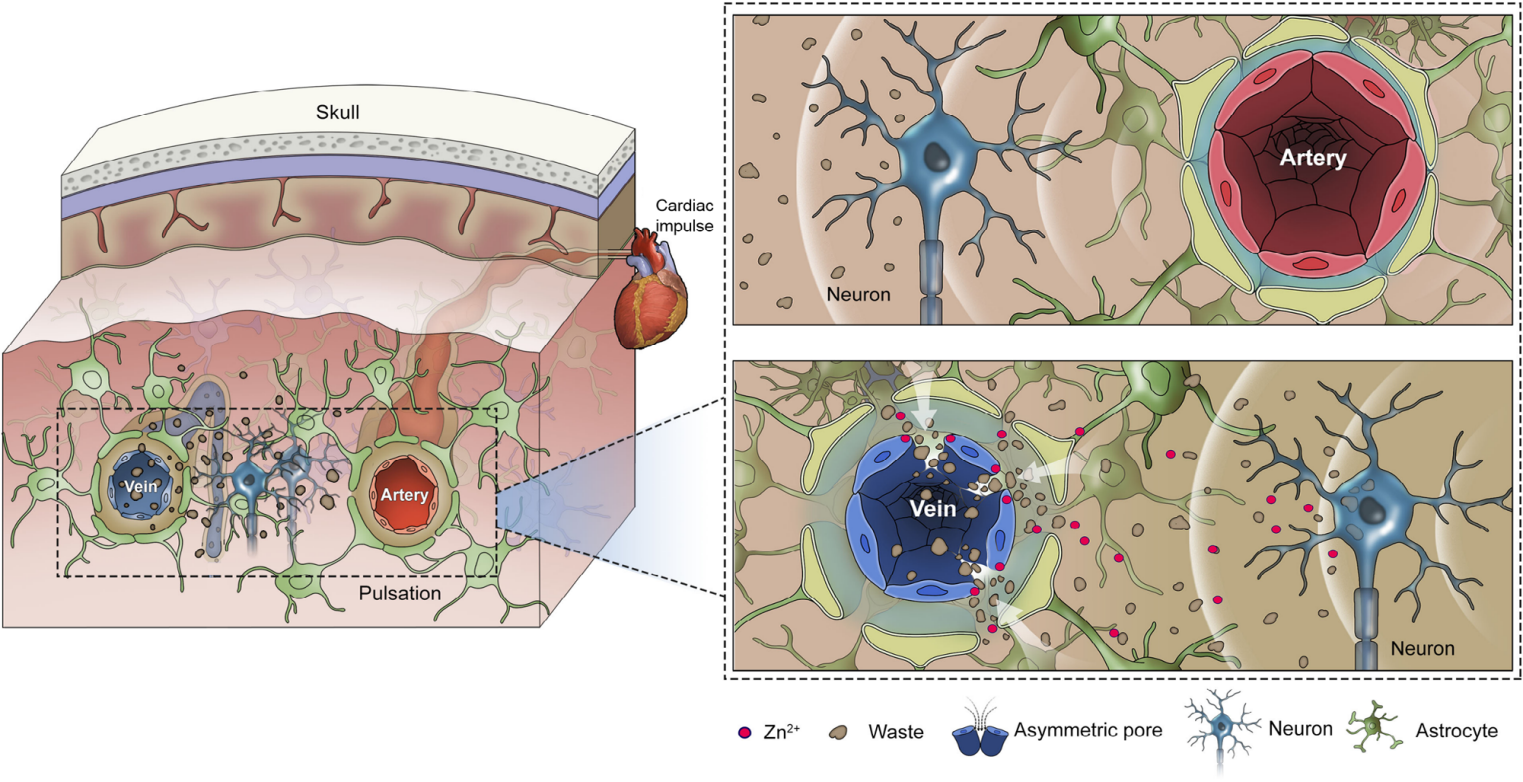
Schematic of updated model for brain clearance of interstitial solutes through the glymphatic system. The actions of astroglial cells primarily during sleep form unique perivascular channels by opening the gaps between vascular endothelial cells and astroglial cells. CSF enters the deep brain following the para‐arterial spaces and takes the interstitial solutes and fluid from the brain parenchyma to the paravenous spaces. Meanwhile, neuronal activity during sleeping CSF oscillation causes release of Zn^2+^ and other regulators to induce remodelling of vascular endothelial cell tight junctions, thus forming the asymmetric pores on venules. Finally, the ISF solutes directly exit into the bloodstream through the dynamic pore paths.

The asymmetric pore paths may provide the final piece for the glymphatic system as an independent pathway coupled with the CSF oscillation for ISF solutes to quickly drain into blood. The updated model is depicted in Figure 5: CSF driven by the wave of CSF oscillation and vascular pulsation follows the periarteriolar spaces into brain parenchyma, collects the interstitial solutes (especially macromolecules and large particles) to the paravenous spaces, and finally exit into bloodstream via the pores on venules induced by neuronal activity. The new mechanism may explain why the activity of glymphatic system is associated with sleep,^[^ ^4^ ^]^ and how most CSF/ISF containing high metabolic wastes is drained out before returning to subarachnoid spaces, thus avoiding cycling of toxic molecules such as Aβ inside brain to stimulate pathogenic processes.

Previous works have suggested inadequate drainage of CSF‐ISF might contribute to the Aβ accumulation in AD.^[^ ^30^, ^31^ ^]^ Hereby, the observations in transgenic AD model mice (Figure 4 and Supplementary Videos 4–6) revealed the interactions between the glymphatic clearance and the pathological factor Aβ. Elevated Aβ is one important aspect of AD pathology recapitulated in the APP/PS1 model.^[^ ^13^, ^36^, ^37^ ^]^ Although the AD mice showed an increased number of pores (Figure 4E), the secretion of high Aβ‐content ISF was observably compromised possibly due to high viscosity of fluid. In addition, although the release of Zn^2+^ stimulated induction of pore formation, it may also aggravate the fluid stickiness/thickness because metal ions such as Cu^2+^, Zn^2+^, Fe^3+^ have been demonstrated to bind to Aβ, thus promoting Aβ aggregation and the formation of insoluble plaque in vitro and in vivo.^[^ ^38^, ^39^ ^]^ The increased retention of Aβ‐content ISF in paravenous space is expected cause alterations in the whole glymphatic system, such as perivascular dilation (even in the upstream section of CSF inflow^[^ ^11^, ^40^, ^41^ ^]^) and Aβ accumulation in the CSF circulation pathway. This may account for Aβ deposition in periarteriolar membranes (rather than perivenular membranes)^[^ ^11^ ^]^ because Aβ peptides showed high affinity to molecules on vascular smooth muscle cells (VSMCs)^[^ ^42^, ^43^ ^]^ such as α7 nicotinic acetylcholine receptor.^[^ ^44^ ^]^

Meanwhile, the sequestration of Zn^2+^ by Aβ plaques would disrupt Zn^2+^ homeostasis, causing a situation of Zn^2+^ deficiency in neural cells but accumulation in brain tissue. The extracellular Zn^2+^ deficiency would further reduce Aβ degradation by decreasing the catalytic activity of several Zn^2+^ metalloproteinases including insulin‐degrading enzyme (IDE), neprilysin (NEP), and matrix metalloproteinases (MMP) etc.^[^ ^45^ ^]^ Therefore, facilitating Zn^2+^ and Cu^2+^ mobility with 8‐hydroxyquinoline derivatives (PBT‐1/PBT‐2) chelate therapy could effectively alleviate AD progress;^[^ ^46^, ^47^ ^]^ Zn supplementation could be help to stabilize the cognitive abilities in AD patients.^[^ ^48^, ^49^, ^50^ ^]^

As the pore path formation requires the viable vascular endothelial cells (VECs), impairment of VEC function would damage the brain ISF solute clearance. For example, impaired VEGF‐C/D–VEGFR3 signalling would reduce clearance of ISF solutes, which may be an alternative or complementary explanation, rather than abrogation of meningeal lymphatic vessels alone. As CSF tracers were not seen passing through arachnoid granulations/villi, suggesting only CSF water drains into the dural venous sinuses, impaired VEGF‐C/D–VEGFR3 would be unlikely to affect CSF water drainage. Nonetheless, any pathological factors affecting VEC function would alter the brain metabolic waste clearance, thus causing neurological damage to the brain. For example, ongoing cognitive deficits and brain atrophy have recently been reported following recovery from COVID‐19, including in those no longer reporting symptoms.^[^ ^51^, ^52^ ^]^ A network‐based, multimodal omics analysis links SARS‐CoV‐2 infection to brain microvascular injury and neuroinflammation;^[^ ^53^ ^]^ In fact, the S1 subunit of the spike protein was shown to cause damage of endothelial cells of micro‐vessels.^[^ ^54^ ^]^ Thus, we speculate that impaired brain waste clearance may play a role in the cognitive impairment like that in AD, and enhancement of the brain glymphatic function while promoting the VEC recovery by certain agents (such as epidermal growth factors, EGFs) are expected to prevent the loss of cognition in COVID‐19 patients.

In summary, we discovered a novel asymmetric pore path on the venule walls for ISF solutes to drain rapidly from brain, which may provide a key final piece for the glymphatic system. The updated model may satisfy all the remaining questions regarding the mechanisms of this clearance process. Full elucidation of the pore structures and regulation mechanisms represent an important target for future studies. Nonetheless, the importance of microvessel endothelial cell function in maintaining the brain metabolic waste clearance and preventing the pathogenesis and progression of a variety of brain diseases should be emphasized.

## CONFLICT OF INTEREST STATEMENT

The authors declare no conflicts of interest.

## Supporting information

Supplemental Video 1

Supplemental Video 2

Supplemental Video 3

Supplemental Video 4

Supplemental Video 5

Supplemental Video 6

Supplemental Video 7

## Data Availability

All data related to this study are present in the article. Any other data associated with this work are available from the corresponding authors upon request.
